# 

**Published:** 1919-11

**Authors:** 


### ANNONCEUMENTS

Ohio State Dental Society. The annual meeting of this society will be held in Columbus, December 2, 3 and 4, 1919, in Memorial Hall. An excellent programme is in preparation and a cordial invitation is extended to members of the profession from other states.

Dental Protective Association. The annual meeting will be held in the Palmer House, Chicago, Monday, December 15th, at 4 p. m. Election of directors and other important business will be transacted. Members are urged to attend.—J. G. Reid, President.

